# Profiles of Growth Factors Secreted by In Vitro-Stimulated Paediatric Acute Leukaemia Blasts of Myeloid and Lymphoid Origin

**DOI:** 10.3390/ijms27020933

**Published:** 2026-01-17

**Authors:** Anna Kozub, Rafał Szarek, Mikołaj Szczęsny, Dagmara Jaworska, Wojciech Młynarski, Jerzy Kowalczyk, Tomasz Szczepański, Zenon P. Czuba, Łukasz Sędek

**Affiliations:** 1 Student Research Group, Department of Microbiology and Immunology, Medical University of Silesia, 40-055 Katowice, Poland; s82907@365.sum.edu.pl (A.K.); rafalszar@vp.pl (R.S.); mikolaj.szczesny@poczta.fm (M.S.); 2 Department of Microbiology and Immunology, Medical University of Silesia, 40-055 Katowice, Poland; djaworska@sum.edu.pl (D.J.); zczuba@sum.edu.pl (Z.P.C.); 3 Department of Paediatrics, Oncology and Haematology, Medical University of Lodz, 90-419 Łódź, Poland; 4 Department of Paediatric Haematology, Oncology and Transplantology, Children’s University Hospital, Medical University of Lublin, 20-093 Lublin, Poland; 5 Department of Paediatric Haematology and Oncology, Medical University of Silesia, 40-055 Katowice, Poland

**Keywords:** colony-stimulating factors, growth factors, G-CSF, GM-CSF, basic FGF, VEGF, PDGF, acute myeloid leukaemia, acute lymphoblastic leukaemia

## Abstract

The research on cytokine or growth factor (GF) release by leukaemic blasts is a largely unexplored area. This study aimed to evaluate the differential secretory potential of paediatric B-cell precursor and T-cell acute lymphoblastic leukaemia (BCP-ALL and T-ALL, respectively) and acute myeloid leukaemia cells (AMLs) for selected GFs, both basally and upon stimulation with phytohemagglutinin (PHA), lipopolysaccharide (LPS), or phorbol 12-myristate 13-acetate with ionophore A23187 (PMA + I). The concentrations of five GFs: granulocyte colony-stimulating factor (G-CSF), granulocyte-macrophage colony-stimulating factor (GM-CSF), basic fibroblast growth factor (b-FGF), vascular endothelial growth factor (VEGF), and platelet-derived growth factor (PDGF) in the supernatants were measured using the Bio-Plex multiplex immunoassay. AML blasts showed the highest basal concentrations of G-CSF, GM-CSF, and VEGF. PHA and LPS stimulation non-selectively enhanced the secretion of G-CSF, GM-CSF, VEGF, and PDGF in BCP-ALL and AML blasts. PMA + I was the strongest GF release inducer, particularly for BCP-ALL and T-ALL blasts, with the latter also showing higher responsiveness to PHA and LPS. Our findings reveal differential, leukaemia-type dependent GF secretion patterns. Lineage-specific responses may be exploitable for targeted therapeutic approaches for distinct AL types. This study is the first to comprehensively assess the extracellular secretion of multiple GFs by paediatric AL cells in cultures using a Bio-Plex multiplex immunoassay.

## 1. Introduction

Acute leukaemias (ALs) are a group of haematological malignancies in which immature cells (blasts) proliferate in an uncontrolled fashion and accumulate in the bone marrow (BM). This excessive growth may involve myeloid or lymphoid precursor cells, resulting in acute myeloid leukaemia (AML) or acute lymphoblastic leukaemia (ALL), respectively. In about 85% of ALL cases, clonal expansion affects the progenitors of B-cells, while T-cell precursor cells are affected in the remaining 15% of diagnoses [1,2]. The majority of childhood AL cases are reported during the first years of life [3]. Overall, it is the most frequent cancer during childhood, accounting for 28% of all paediatric tumours [4]. In recent years, the prognosis has markedly advanced and paediatric survival rates currently achieve >90% in ALL and approximately 70% in AML diagnosis [5,6]. Predisposition to AL is complex, encompassing genetic disorders and syndromes, environmental factors, or previous treatment administration, among others [7].

The ability of leukaemic cells to secrete particular types of cytokines or growth factors (GF) in cultures, especially upon mitogenic stimulation, has not been previously explored. It is, however, clearly evidenced that the BM and peripheral blood (PB) of children with AL ‘naturally’ exhibit a complex network of these substances. They may be one of the key factors in understanding the aetiopathogenesis of AL [8,9,10,11]. For example, cytokine networks altered in AL, including transforming growth factor-β-dependent signalling and extracellular vesicle-mediated communication contribute to BM niche dysregulation [12,13]. It is suggested that the presence of leukaemic cells may introduce an immune imbalance between pro- and anti-inflammatory profiles of the BM microenvironment [14,15]. It has also been shown that blast cells in B-cell precursor acute lymphoblastic leukaemia (BCP-ALL) can take control of the BM microenvironment. This may, among others, manifest in the production of chemotherapy-induced cytokines, which facilitates chemoresistance [16,17]. On the contrary, the BM microenvironment may also have a modulating effect on the metabolism and protein expression of ALL cells [18]. In vitro co-culture studies have demonstrated that a bidirectional cross-talk between BM stromal cells (including fibroblasts and osteoblasts) and lymphoid and myeloid precursor cells can occur, impacting cell responsiveness to different stimuli, cellular signalling, proliferation, or protein synthesis [19,20,21]. Thus, the altered cytokine concentrations may either underlie the pathogenesis of AL or result from modulation of the BM microenvironment by leukaemic blasts.

Studies focused on the GF profile of paediatric AL are scarce in the published literature, although some of them are known to influence the maturation and proliferation of leukaemic cells of different types [22]. The most clinically important GFs, with a well-grounded long-term impact on remission prevention and the enhancement of overall survival rates of children, especially with ALL, are the granulocyte colony-stimulating factor (G-CSF) and granulocyte-macrophage colony-stimulating factor (GM-CSF) [23,24]. These GFs are used as supportive chemotherapeutic agents in different haematological disorders, including ALL, and improve the recovery of neutrophils and/or monocytes after haematopoietic stem cell transplantation, reduce the risk of neutropenia and infection, and increase the sensitivity of leukaemic cells to cytotoxic drugs in many therapeutic protocols [22,23,24,25]. On the other hand, it is proven that these GFs can also stimulate the proliferation of residual leukaemic cells of myeloid origin, thus their usage in AML is currently restricted [26].

Basic fibroblast growth factor (b-FGF), platelet-derived growth factor (PDGF), and vascular endothelial growth factor (VEGF) have not been widely studied in the context of their potential influence on leukaemic cells or their possible therapeutic use. Single studies show that FGF may stimulate the growth of leukaemic blast colonies, but with a less pronounced efficacy than that of G-CSF and GM-CSF [27] and that PDGF may stimulate proliferation of the blast cultures derived from patients with AML [28]. In turn, elevated levels of VEGF family members (VEGF-A) in the cerebrospinal fluid of ALL patients were observed in patients with confirmed central nervous system infiltration [29,30].

In our study, we aimed to investigate the differential secretory potential of leukaemic blasts from paediatric patients with ALL and AML, measured as their ability to produce the selected growth factors: G-CSF, GM-CSF, b-FGF, VEGF, and PDGF. We also tried to determine the possible additive role of stimulation with mitogens such as phytohemagglutinin (PHA) and lipopolysaccharide (LPS), or other cell activators, such as phorbol 12-myristate 13-acetate together with ionophore A23187 (PMA + I).

## 2. Results

### 2.1. Determination of the Influence of the Dissolvent on Cell Stimulation

**Figure 1:**
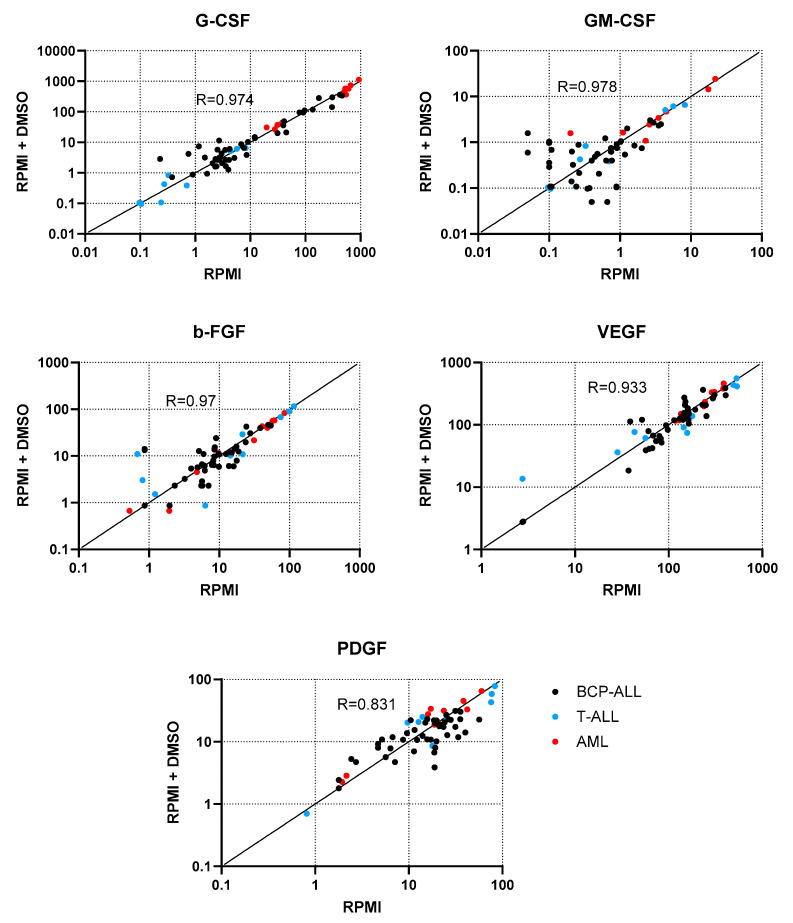
Concentration of the examined growth factors in control samples with pure RPMI vs. RPMI supplemented with <0.1% DMSO. Data points corresponding to BCP-ALL, T-ALL, and AML are shown in black, blue, and red, respectively. The Pearson’s correlation coefficient R values are shown in respective plots.

We demonstrated that the GF levels between control samples in which the cells were suspended in pure RPMI vs. those suspended in RPMI supplemented with DMSO were virtually the same. The mean Pearson’s correlation coefficient for all tested stimulators was 0.94 (range 0.83–0.97; Figure 1). This indicates that DMSO in a concentration of <0.1% present in all test samples with stimulators did not influence the GF concentration obtained in stimulated samples.

### 2.2. Basal Concentrations of the Examined GF

**Figure 2:**
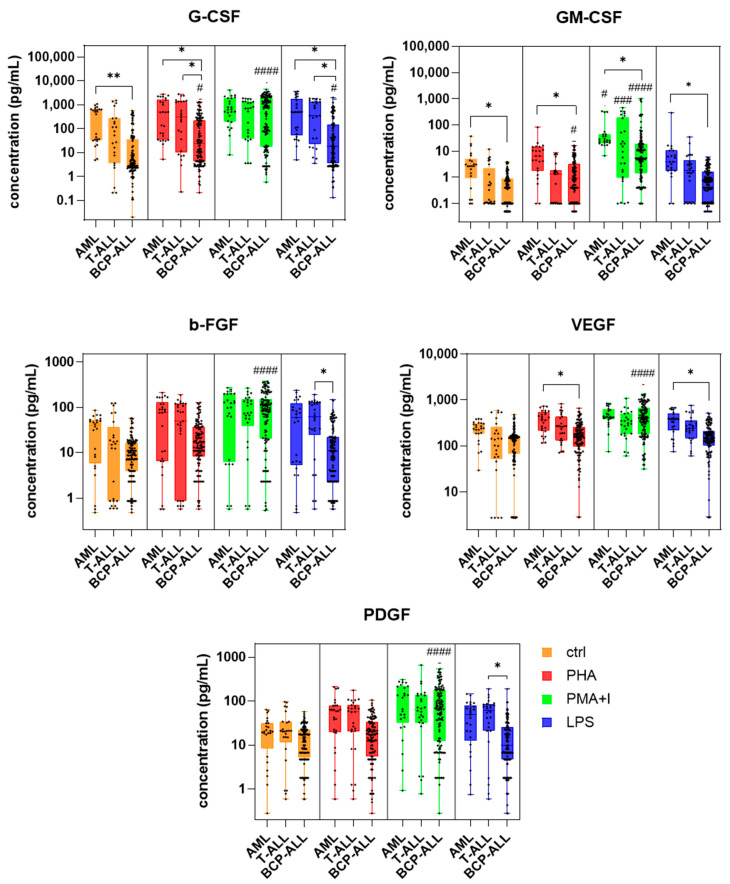
Concentration of the examined growth factors in control and PHA-, PMA + ionophore A23187 (PMA + I)-, and LPS-stimulated samples of BCP-ALL, T-ALL, and AML cells. Significant differences between stimulated vs. control conditions were marked with hash symbols (# *p* < 0.05, ### *p* < 0.001, #### *p* < 0.0001). Significant differences between different leukaemia types were marked with asterisks (* *p* < 0.05, ** *p* < 0.01).

In unstimulated conditions, AML blasts exhibited the highest median concentrations of G-CSF, GM-CSF, b-FGF, and VEGF, which reached 454.9 pg/mL, 2.7 pg/mL, 40.0 pg/mL, and 240.1 pg/mL, respectively (Table 1, Figure 2). Basal levels of these four GFs were generally lower in T-ALL and BCP-ALL samples, but significantly lower values were only noted between BCP-ALL and AML blasts for G-CSF and GM-CSF (120-fold lower, *p* = 0.0013, and 7-fold lower, *p* = 0.0153, respectively). The median concentration of PDGF across all leukaemia types was not significantly different and ranged between 14.2 and 20.9 pg/mL (Table 1, Figure 2).

### 2.3. Response of Leukaemic Cells to Stimulation

#### 2.3.1. G-CSF

The highest (almost 140-fold higher than the control) increase in median G-CSF level was observed for PMA + I stimulation of BCP-ALL blasts (median G-CSF concentration of 500.2 pg/mL, *p* < 0.0001). Stimulation of BCP-ALL cells with PHA and LPS also resulted in a significant, but more moderate (around 5–6.5-fold) increase in the median G-CSF level, as compared to the control conditions (*p* = 0.028 and *p* = 0.038, respectively). The median level of G-CSF in the stimulated T-ALL samples was 6–13.5 times higher than in control conditions; however, the statistical significance was not reached for any of the stimulators used.

#### 2.3.2. GM-CSF

The release of this GF was strongly enhanced by PMA + I in all leukaemic blast types, with the strongest (over 88-fold) increase vs. the control observed in T-ALL (12.4 pg/mL, *p* = 0.0005). For the BCP-ALL and AML samples, over a 14-fold (5.7 pg/mL, *p* < 0.0001) and over 9-fold (25.5 pg/mL, *p* = 0.0199) increase in median GM-CSF levels was observed, respectively. Besides PMA + I, BCP-ALL blasts were also significantly stimulated by PHA, which increased the median GM-CSF level around 2-fold vs. the control (0.89 pg/mL, *p* = 0.0219). Moreover, we observed a moderate stimulating effect of LPS on T-ALL blasts, which increased the median GM-CSF level around 7 times vs. the control (0.94 pg/mL), but this difference was not significant (*p* = 0.1009).

#### 2.3.3. b-FGF

A significant, almost a 10-fold increase in median b-FGF level was observed in BCP-ALL samples on stimulation with PMA + I (89.2 pg/mL, *p* < 0.0001). PMA + I also stimulated b-FGF release by T-ALL blasts, in which around a 5-fold increase was observed, as compared to control conditions (75.0 pg/mL), but without reaching statistical significance (*p* = 0.0642). AML cells were not significantly stimulated by any of the stimulators used. We also did not observe any significant stimulatory effect of PHA and LPS on b-FGF production in any type of leukaemic blasts.

#### 2.3.4. VEGF

Alike GM-CSF, a significant but mild increase in median levels of VEGF in all leukaemic cell types was only observed after stimulation by PMA + I. In BCP-ALL cells, PMA + I caused about a 2.7-fold increase in median VEGF level as compared to control (397.5 pg/mL, *p* < 0.0001). In T-ALL and AML samples, about a 2-fold increase was observed; however, this was not significant as compared to the control (307.1 pg/mL, *p* = 0.0563 and 432.3 pg/mL, *p* = 0.0563, respectively). We did not observe any significant effects of PHA and LPS on the production of VEGF by any leukaemic cell type.

#### 2.3.5. PDGF

The increased production of PDGF was only observed for BCP-ALL blasts upon PMA + I stimulation, which increased the median PDGF level around 5-fold in BCP-ALL (68.7 pg/mL, *p* < 0.0001). For AML and T-ALL samples, an almost 6-fold and a 3-fold increase in PDGF production was observed, as compared to the control conditions, but statistical significance vs. the control was not reached (119.4 pg/mL, *p* = 0.0896 and 63.9 pg/mL, *p* = 0.103, respectively). No significant stimulating effect of PHA and LPS on PDGF production was observed in any leukaemic cell type.

### 2.4. Lineage-Specific Responses to Stimulation

The basal advantage in median G-CSF and GM-CSF production by AML vs. BCP-ALL blasts was also maintained after PHA and LPS stimulation and after PMA + I stimulation for GM-CSF only (Table 1, Figure 2). Concerning VEGF levels, even though at unstimulated conditions no significant differences were visible between different leukaemia types, PHA and LPS stimulation revealed substantial differences between AML and BCP-ALL blasts (375.4 pg/mL vs. 158.5 pg/mL, *p* = 0.0135, for PHA stimulation, respectively and 381.9 pg/mL vs. 149.1 pg/mL, *p* = 0.023, for LPS stimulation, respectively), as was also seen for G-CSF and GM-CSF. This indicates that stimulation with PHA and LPS non-selectively enhances the secretion of these GFs by both BCP-ALL and AML cells.

Some significant differences between different leukaemia types were exhibited, even though they were not all visible in the unstimulated state. For example, significantly higher levels of G-CSF, b-FGF, and PDGF were observed in LPS-stimulated T-ALL samples, as compared to BCP-ALL (348.9 pg/mL vs. 18.8 pg/mL, *p* = 0.0154 for G-CSF, 61.7 pg/mL vs. 11.0 pg/mL, *p* = 0.0397 for b-FGF and 62.3 pg/mL vs. 9.7 pg/mL, *p* = 0.0104 for PDGF; Table 1, Figure 2). This observation would support a more selective, cell-lineage-dependent impact of LPS on GF secretion. Moreover, for PHA-stimulated cells, a significantly higher level of G-CSF was observed in T-ALL, as compared to BCP-ALL cells (298.6 pg/mL vs. 23.9 pg/mL, respectively, *p* = 0.028).

## 3. Discussion

The native ability of leukaemic cells to produce immunoregulatory factors such as cytokines or GFs in in vitro cultures represents an unexplored field at the border of clinical haematology and immunology. Moreover, their ability to respond to various chemical stimuli also remains largely unknown. In this pilot study, we investigated the secretion profiles of selected GFs shown by AML, T-ALL, and BCP-ALL leukaemic blasts without stimulation, followed by the incubation with that commonly used mitogens PHA and LPS, and PMA with ionophore A23187, which are known to promote cell growth and differentiation [31].

Undoubtedly, the presence of mitogens or other activators in cell cultures results in the acceleration of cell proliferation. In the context of cultured AL cells, important observations were already made in some dated studies, but only a few examples of similar studies exist in contemporary literature. Sumer et al. studied the blastogenic response of PB MNC (containing both lymphocytes and blasts) collected from ALL patients at different stages of the therapy, including complete remission. The authors used PHA stimulation in MNC cultures and recorded the time at which the maximum proliferative response occurred. It was found that early PHA-responders had significantly worse disease-free interval and survival rates than the late responders. The authors concluded that the early PHA-responders were the patients with higher blast vs. normal lymphocyte load, as compared to the late responders. This suggests that the response of normal lymphocytes and leukaemic blasts, particularly those of the T-cell phenotype, to the mitogen is different, and the time of response could be used indirectly as a measure of PB blast infiltration, thus providing important prognostic value [32]. Regarding AML, in a study performed by Swart et al., AML blasts collected from both BM and PB samples were cultured on PHA-supplemented and standard leukocyte feeder layers. The results revealed that the PHA leukocyte feeder cultures consisted mostly of blast cells. In contrast, in standard leukocyte feeder cultures, macrophages and monocytes were detected, suggesting that PHA prevented blast maturation into mature myeloid cells [33]. Even though measurement of cytokine and/or GF level was not performed in the study, an indirect conclusion can be made that PHA might contribute to the secretion of some cytokines or GFs (like G-CSF or GM-CSF) that indeed accelerate blasts’ proliferation, but hamper their maturation into more mature cells of myeloid lineage. This assumption is in line with other observations that G-CSF or GM-CSF can stimulate the proliferation of not only normal myeloid progenitors but also the residual abnormal leukaemic cells [26]. This effect of GM-CSF and G-CSF was also observed in in vitro studies with the blast cells isolated from ALL and AML patients [34,35,36]. In a newer study on BM samples collected from childhood ALL patients, it was shown that PHA stimulation caused colony formation in the majority of samples, emphasising the ability of PHA to stimulate cell division, compared to the unstimulated samples [37].

Stimulation of PB MNC cells of ALL patients with PHA was also examined by Ridgway et al. The authors have shown that the production of GM-CSF and IL-1 by the PHA-stimulated PB MNCs of paediatric ALL patients receiving maintenance chemotherapy was diminished as compared to the control, suggesting that PHA may damage key cytokine-production mechanisms [38]. The diminished ability to produce GM-CSF and IL-1 may, in turn, contribute to the increased infection susceptibility in ALL patients [38,39].

Only a few studies on the cytokines or GFs secreted by cultured blast cells derived from children with AL, which would specifically have been conducted with Bio-Plex Luminex technology, exist in the published literature. In the majority of studies involving human AL cells, cytokines were examined mainly in BM/PB serum or other materials from ALL patients, e.g., saliva [10,11,30,40,41,42,43]. In one of such studies examining the cytokine and GF concentrations in the PB of children treated for ALL, it was found that the levels of 33 of the 51 examined cytokines and GFs, including GM-CSF, G-CSF, VEGF, and PDGF, were significantly higher as compared to healthy children, while no significant differences were observed for b-FGF levels [42]. Nevertheless, direct reference of our results to this type of finding is difficult and susceptible to speculation.

In our experiment series, stimulation with PHA, as well as with LPS, boosted the secretion of G-CSF, GM-CSF, and VEGF by both BCP-ALL and AML blasts and deepened the basal difference between these cell types in favour of AML blasts. We also demonstrated that PHA, but also LPS, significantly increased the levels of G-CSF in BCP-ALL blasts vs. the control (up to 23.9 pg/mL for PHA and 18.8 pg/mL for LPS stimulation vs. 3.7 pg/mL for the control; *p* = 0.028 and *p* = 0.0376, respectively). Since G-CSF can be of potential therapeutic use, especially in ALL, the administration of PHA to these patients and the stimulation of G-CSF production by leukaemic blasts might be beneficial. Such trials were recently performed by Alhallak et al., who encapsulated PHA in liposomes, thus increasing its stability and reducing its toxicity. In this way, PHA becomes a candidate molecule to circumvent current problems related to T-cell-based immunotherapies, such as the relatively short half-life of ex vivo T-cell activation and T-cell exhaustion [44]. PHA in liposome form could potentially be used not only as an alternative method of T-cell activation for immunotherapeutic purposes, but also for the stimulation of GF production by ALL cells. Moreover, we showed that PHA stimulation caused even higher stimulation of T-ALL vs. BCP-ALL blasts, which resulted in a significantly higher level of G-CSF secreted by T-ALL blasts (298.6 pg/mL vs. 23.9 pg/mL, respectively, *p* = 0.0128). This points to selective sensitivity of T-lineage cells to PHA, which is known to act specifically as a T-cell mitogen [32]. It can be assumed that PHA in liposome form might also be useful in T-ALL therapy as well.

Higher responsiveness of T-ALL vs. BCP-ALL blasts was also revealed by LPS stimulation, which resulted in significantly higher levels of three out of five of the examined GFs secreted by T-ALL blasts, while, without any stimulation, the differences between both lineages were insignificant. Supportive data in the literature explaining this phenomenon were unfortunately not found. Contrastingly, it was suggested that LPS more specifically induced cytokine production in monocytes, rather than in T-cells, when measured intracellularly [45]. A study on LPS was also performed by Assaf et al., who cultured AL blasts with a flavonoid cyanidin-3-glucoside (C3G) in the presence or absence of LPS. It was shown that C3G decreased the levels of several cytokines and reduced migration ability, especially in cells challenged by LPS [46]. LPS-enhanced inhibition of migration seems to be of particular importance as it is capable of diminishing leukaemic cell dissemination to extramedullary sites. LPS was also used to stimulate the cell differentiation of leukaemic cell models of sepsis [47].

PMA is known to be capable of driving cell differentiation, proliferation, and cytokine production, particularly by T-cells [45]. It was suggested that PMA can mimic the action of diacylglycerol, binding to and activating protein kinase C-dependent signalling cascades, which are crucial for the induction of NFAT and AP-1 and the initiation of the transcription of various cytokine genes [31,48]. Concerning the leukaemic cells, several studies also confirmed that PMA induced AML and T-ALL cells to produce and/or activate transcription factors and other important proteins related to differentiation [49,50,51,52,53,54]. In turn, in a study of Kharfan-Dabaja et al., PMA caused AML blast differentiation toward a dendritic cell-like phenotype, with increased expression of co-stimulatory molecules such as CD80 and CD86 [55]. Our findings showed that PMA (together with the ionophore A23187) strongly induced, particularly BCP-ALL cells, to produce all of the examined GFs, which reached up to 140-fold higher concentrations, as compared to the control (500.2 pg/mL for G-CSF, 397.5 pg/mL for VEGF, 89.2 pg/mL for b-FGF, 68.7 pg/mL for PDGF and 5.7 pg/mL for GM-CSF vs. 3.7 pg/mL for the control, all *p* < 0.0001). Spectacular PMA + I stimulation was also observed for T-ALL blasts, resulting in an over 88-fold increase in GM-CSF levels (12.4 pg/mL vs. 0.14 pg/mL, respectively, *p* = 0.0005). AML cells were stimulated to a lesser degree and mainly to produce GM-CSF (25.5 pg/mL vs. 2.7 pg/mL for the control, *p* = 0.0199). This overall strong stimulatory effect of PMA + I lacks cell-lineage specificity, which might hamper its potential use in targeted therapies of different leukaemia types. Furthermore, PMA was reported to have a carcinogenic potential [56], which would need to be overcome in any in vivo studies.

The possible therapeutic use of b-FGF, PDGF, and VEGF and their potential influence on leukaemic cells has not been extensively studied. It was suggested that FGF may stimulate blast colony growth; however, its effect was less pronounced than that of G-CSF and GM-CSF [27]. Similarly, a limited impact of PDGF on proliferation was demonstrated in part of the blast cultures derived from AML patients, which was dependent on both the PDGF isoform and the presence of G-CSF and GM-CSF [28]. Regarding VEGF family members, a study conducted on children with ALL, including nine patients with confirmed central nervous system localisation (CNSL), demonstrated that the BM levels of VEGF-A at the beginning and in further therapy stages were similar and that the reduction of leukaemic blasts in BM did not lead to diminished VEGF-A levels. Moreover, it was suggested that an elevated VEGF-A level in cerebrospinal fluid (CSF) samples is associated with CNSL [29,30].

Despite the prognostic potential of these results, we cannot directly relate all of them to our findings due to the different methodologies used. Nevertheless, all of these findings emphasise the significance of different approaches aiming for the determination of GF levels not only in PB, BM, or CSF, but also those in in vitro cultured cells, which altogether may deliver potentially important clinical information. Our findings highlight differential, leukaemia-type dependent GF secretion patterns, reflected by their differential levels both in basal conditions and upon stimulation with the examined stimulators. We suppose that the cell-lineage specificity may be exploitable for a possible targeted therapeutic approaches for distinct AL types (i.e., AML, T-ALL, BCP-ALL), particularly PHA administered in encapsulated form, stimulating G-CSF production. While our findings provide novel insights, the lack of comparable studies highlights the need for further, more robust research on GF or other cytokines to draw definitive conclusions on the meaning of the obtained results.

To conclude, our study has developed the current state of knowledge on the profiles of secretion of several GFs without or with the addition of common cell stimulators. To our knowledge, this is the first study to comprehensively evaluate the secretion of multiple GFs produced by paediatric AL cells in cultures, measured extracellularly with the application of the Bio-Plex multiplex immunoassay. The selected method was proven to be a sensitive and practical approach, suitable especially for small-volume clinical samples. We are aware of several limitations of the performed study. One of these is the possible contamination of MNC samples with residual mature lymphocytes; however, taking into account that the initial blast load exceeded 75% in every full BM sample, this influence should not be significant. However, this bystander cell effect cannot be reliably estimated, therefore future studies should take this effect into account by performing leukaemic cell extraction, rather than enrichment, as was performed in this study. For additional reference, BM samples collected from healthy children could also be collected, but, due to the difficulty in accessing such samples, this was not performed. Another option might also include the collection of other materials, i.e., PB or saliva, which would at least give an indirect insight into the GF levels within the healthy control group. Our results may additionally be biased by the imbalanced size of the study groups: BCP-ALL (*n* = 46) vs. T-ALL (*n* = 10) vs. AML (*n* = 10), with a relatively low sample size in the two latter groups, translating into low statistical test power assessed post hoc. This fact could potentially have hampered the discovery of true differences between the secretion of GFs by the three leukaemic cell types.

## 4. Materials and Methods

### 4.1. Patients

The study group consisted of 66 children (33 males, 33 females) with a median age of 5.2 years, with newly confirmed BCP-ALL (*n* = 46), T-ALL (*n* = 10), or AML (*n* = 10) before the start of the therapy. The patients were treated in 16 centres belonging to the Polish Paediatric Leukaemia and Lymphoma Study Group. BM samples were shipped to the central laboratory of the Medical University of Silesia in Katowice, located in Zabrze, for immunophenotyping with the use of flow cytometry (FC) in the period of November 2023–September 2024. The leftovers of the BM samples remaining after the diagnostic process were subjected to subsequent blast cell isolation.

### 4.2. Leukaemic Cell Enrichment

The leftover BM samples were diluted in phosphate-buffered saline (PBS; buffer solution, pH ~7.4, PAN Biotech, Aidenbach, Germany) and subjected to density gradient centrifugation at 400× *g* for 30 min, using Histopaque 1077 (Sigma-Aldrich, St. Louis, MO, USA) in 15 mL test tubes. The initial percentage of blasts in the full BM samples was determined by FC and ranged between 75 and 95% of total cells. The mononuclear cell (MNC) layer containing the blast cells was subsequently transferred with a Pasteur pipette to new test tubes and washed with PBS by centrifugation at 540× *g* for 5 min. Afterwards, the supernatants were discarded and the blast cells were diluted in 1 mL of RPMI medium (no. 1640, ATCC, Manassas, VA, USA). The number of blasts was estimated manually in the Bürker chamber. Next, the blasts were cultured at an estimated concentration of 1 million cells per mL for 48 h in several sterile culture bottles (Corning, Corning, NY, USA) and incubated at 37 °C. For AML, all 10 samples in all conditions were measured in 3 replicates, for T-ALL, 9/10 samples were measured in 3 replicates and 1/10 in 4 replicates. For the BCP-ALL samples, 9/46 samples were measured in 2 replicates, 34/46 samples in 3 replicates, and 2/46 samples in 4 replicates. A single replicate contained 5 million cells.

### 4.3. Cell Stimulation

After 48 h of culture, the total content of the culture bottles was transferred under a laminar flow cabinet to the test tubes and centrifuged. The blasts were subsequently resuspended in fresh RPMI medium and seeded into culture plates in an amount of 0.5 mL per well. The wells were then filled up to 1 mL with either pure RPMI medium or a respective cell stimulator solution in RPMI, to achieve a final concentration of 1 million blasts in 1 mL of medium.

The following two mitogens: phytohemagglutinin (PHA; Difco, Detroit, MI, USA) and lipopolysaccharide (LPS; Escherichia coli serotype O111, Sigma-Aldrich, St. Louis, MO, USA), and cell stimulator phorbol 12-myristate 13-acetate (PMA; Sigma-Aldrich, St. Louis, MO, USA) together with the ionophore A23187 (I; Sigma-Aldrich, St. Louis, MO, USA), were used, further referred to as as stimulators. Stock solutions of PHA, LPS, and PMA + I in DMSO (Sigma-Aldrich, St. Louis, MO, USA) were serially diluted in RPMI to reach the working concentrations of 10 µg/mL, 1 µg/mL, and 50 ng/mL + 1 µg/mL, respectively.

Samples in which the cells were suspended in pure RPMI served as a control. To eliminate the potential influence of DMSO on cell stimulation, we used additional controls in which the cells were suspended in RPMI supplemented with DMSO in a concentration not exceeding 0.1%, reflecting the amount of DMSO in which the stimulators were primarily dissolved and further used in the test conditions. All plates were incubated for 24 h at 37 °C and then centrifuged at 1000 rpm for 10 min. The supernatants were collected in Eppendorf test tubes and frozen at −20 °C until the Bio-Plex examination.

### 4.4. Growth Factor (GF) Immunoassay

The GF concentrations were measured using the Bio-Plex 3D Suspension Array System (Bio-Rad Laboratories Inc., Hercules, CA, USA), and analysed with Bio-Plex Manager Software version 6.0. Just before the examination, the supernatants were thawed, centrifuged at 12,500 rpm for 5 min, and then approximately 15 µL of every clear fluid obtained was transferred to dedicated 96-well plates. A certified immunoassay kit (BioPlex Human Cytokine Panel, Bio-Rad Laboratories Inc., Hercules, CA, USA) profiled for the examined cytokines was used.

### 4.5. Statistical Analysis

Normality of the results distribution was checked with the Shapiro–Wilk test. Since the measured GF concentrations exhibited non-normal distribution, Kruskal–Wallis ANOVA was used, followed by the two-stage linear step-up procedure of Benjamini, Krieger, and Yekutieli. This procedure was used to control the false discovery rate associated with the use of multiple comparisons. Adjusted *p*-values were gathered in Supplementary Table S1 and values < 0.05 were considered statistically significant. Since part of the GF concentrations in different conditions were measured with only 2 replicates, additional sensitivity analysis excluding these measurements was performed, and the adjusted *p*’-values were collected in Supplementary Table S2. Moreover, we performed post hoc power analysis to show the impact of relatively low subgroup sizes for T-ALL and AML (*n* = 10). The results are collected in Supplementary Table S3.

To determine the correlation of the data, Pearson’s correlation coefficient was calculated. *p*-values < 0.05 were considered significant. Statistical analyses were performed with Statistica software v. 13 (Tibco Software, Tulsa, OK, USA), GraphPad Prism v. 10.4.2 (GraphPad Software, Boston, MA, USA), and G*Power v. 3.1.9.7 software (Franz Faul, Universität Kiel, Germany) [57]. For data visualisation, GraphPad Prism was used.

## Figures and Tables

**Table 1:** Median concentrations of the examined growth factors in cultures of leukaemic blasts of BCP-ALL, T-ALL, and AML patients.

Growth Factor	Basal/ Stimulated Conditions	AML	T-ALL	BCP-ALL
		Median Concentration (IQR) [pg/mL]		
G-CSF	RPMI	454.87 (596.82)	52.18 (245.17)	3.65 (1.74)
	RPMI + DMSO	475.93 (582.12)	27.05 (273.72)	3.96 (13.05)
	PHA	482.15 (1443.68)	298.64 (1370.42)	23.94 (217.08)
	PMA + I	614.55 (1621.03)	702.12 (1309.62)	500.24 (1867.88)
	LPS	496.76 (1581.73)	348.85 (1310.8)	18.75 (144.54)
GM-CSF	RPMI	2.7 (3.3)	0.14 (0.87)	0.4 (0.81)
	RPMI + DMSO	2.42 (2.7)	0.11 (0.67)	0.4 (0.67)
	PHA	6.52 (12.65)	1.31 (1.71)	0.89 (3.05)
	PMA + I	25.46 (23.4)	12.36 (190.95)	5.66 (16.97)
	LPS	3.96 (8.79)	1.85 (3.36)	0.75 (1.49)
basic FGF	RPMI	39.98 (46.97)	14.89 (22.0)	9.07 (12.13)
	RPMI + DMSO	38.07 (46.89)	10.82 (30.5)	9.96 (11.46)
	PHA	77.12 (122.78)	49.20 (119.66)	15.61 (28.08)
	PMA + I	124.09 (191.19)	75.01 (110.85)	89.16 (131.03)
	LPS	59.78 (105.8)	61.67 (98.99)	10.99 (19.55)
VEGF	RPMI	240.12 (122.87)	136.05 (149.43)	147.21 (97.4)
	RPMI + DMSO	221.05 (177.88)	74.53 (92.52)	130.57 (108.49)
	PHA	375.36 (293.75)	264.12 (266.69)	158.47 (161.4)
	PMA + I	432.3 (225.51)	307.07 (259.52)	397.5 (490.74)
	LPS	381.85 (278.29)	241.85 (190.55)	149.09 (110.59)
PDGF	RPMI	20.15 (23.05)	20.85 (22.25)	14.22 (15.96)
	RPMI + DMSO	26.27 (13.67)	20.20 (29.31)	11.84 (16.2)
	PHA	58.6 (59.27)	56.50 (55.00)	17.01 (7.68)
	PMA + I	119.38 (173.99)	63.87 (104.5)	68.72 (163.36)
	LPS	48.56 (62.90)	62.28 (62.85)	9.66 (19.23)

## Data Availability

The datasets presented in this article are not readily available because the data are part of an ongoing study. Requests to access the datasets should be directed to the corresponding author.

## Supplementary Materials

The following supporting information can be downloaded at https://www.mdpi.com/article/10.3390/ijms27020933/s1.

## Abbreviations

The following abbreviations are used in this manuscript:

GF	Growth factor
BCP-ALL	B-cell precursor and T-cell acute lymphoblastic leukaemia
T-ALL	T-cell acute lymphoblastic leukaemia
PHA	phytohemagglutinin
LPS	lipopolysaccharide
AML	acute myeloid leukaemia
PMA + I	phorbol 12-myristate 13-acetate with ionophore A23187
GM-CSF	granulocyte-macrophage colony-stimulating factor
G-CSF	granulocyte colony-stimulating factor
b-FGF	basic fibroblast growth factor
VEGF	vascular endothelial growth factor
PDGF	platelet-derived growth factor
MNC	mononuclear cell
DMSO	dimethyl sulfoxide
CNSL	central nervous system localisation
CSF	cerebrospinal fluid
