# Supplementary material for: Profiles of Growth Factors Secreted by In Vitro-Stimulated Paediatric Acute Leukaemia Blasts of Myeloid and Lymphoid Origin
Source: Int J Mol Sci. 2026 Jan 17;27(2):933. doi: 10.3390/ijms27020933 (PMC12841711; doi:10.3390/ijms27020933)
Supplement: Supplementary file 1 [file ijms-27-00933-s001.zip › Supplementary Table S1.pdf]

Supplementary Table S1. Adjusted p values after application of the Benjamini, Krieger and Yekutieli procedure to control the false discovery rate associated with multiple comparisons of concentrations of the examined growth factors in cultures of leukaemic blasts of BCP-ALL, T-ALL and AML patients. Significant differences between stimulated vs. control conditions were marked with hash symbols (# p<0.05, ### p<0.001, #### p<0.0001). Significant differences between different leukaemia types were marked with asterisks (* p<0.05, ** p<0.01). Parts of the table containing the data for specific growth factor were truncated to only show 21 essential comparisons used for drawing conclusions of the performed study.

GM-CSF

All cases

Compared groups	Adjusted p value	Significance level
AML ctrl vs. BCP-ALL ctrl	0.0153	*
AML ctrl vs. T-ALL ctrl	0.0864	
T-ALL ctrl vs. BCP-ALL ctrl	0.49	
BCP-ALL PHA vs. BCP-ALL ctrl	0.0219	#
BCP-ALL PMA+I vs. BCP-ALL ctrl	<0.0001	####
BCP-ALL LPS vs. BCP-ALL ctrl	0.1717	
T-ALL PHA vs. T-ALL ctrl	0.3203	
T-ALL PMA+I vs. T-ALL ctrl	0.0005	###
T-ALL LPS vs. T-ALL ctrl	0.1009	
AML PHA vs. AML ctrl	0.3222	
AML PMA+I vs. AML ctrl	0.0199	#
AML LPS vs. AML ctrl	0.3886	
BCP-ALL PHA vs. T-ALL PHA	0.6272	
BCP-ALL PMA+I vs. T-ALL PMA+I	0.1866	
BCP-ALL LPS vs. T-ALL LPS	0.1007	
BCP-ALL PHA vs. AML PHA	0.0337	*
BCP-ALL PMA+I vs. AML PMA+I	0.0476	*
BCP-ALL LPS vs. AML LPS	0.0153	*
T-ALL PHA vs. AML PHA	0.1009	
T-ALL PMA+I vs. AML PMA+I	0.361	
T-ALL LPS vs. AML LPS	0.3279	

G-CSF

All cases

Compared groups	Adjusted p value	Significance level
AML ctrl vs. BCP-ALL ctrl	0.0013	**
AML ctrl vs. T-ALL ctrl	0.3659	
T-ALL ctrl vs. BCP-ALL ctrl	0.0539	
BCP-ALL PHA vs. BCP-ALL ctrl	0.028	#
BCP-ALL PMA+I vs. BCP-ALL ctrl	<0.0001	####
BCP-ALL LPS vs. BCP-ALL ctrl	0.0376	#
T-ALL PHA vs. T-ALL ctrl	0.246	
T-ALL PMA+I vs. T-ALL ctrl	0.1697	
T-ALL LPS vs. T-ALL ctrl	0.1928	
AML PHA vs. AML ctrl	0.5717	
AML PMA+I vs. AML ctrl	0.3716	

AML LPS vs. AML ctrl	0.5717	
BCP-ALL PHA vs. T-ALL PHA	0.028	*
BCP-ALL PMA+I vs. T-ALL PMA+I	0.5717	
BCP-ALL LPS vs. T-ALL LPS	0.0154	*
BCP-ALL PHA vs. AML PHA	0.0149	*
BCP-ALL PMA+I vs. AML PMA+I	0.3021	
BCP-ALL LPS vs. AML LPS	0.0119	*
T-ALL PHA vs. AML PHA	0.6655	
T-ALL PMA+I vs. AML PMA+I	0.5813	
T-ALL LPS vs. AML LPS	0.6703	

b-FGF

All cases

Compared groups	Adjusted p value	Significance level
AML ctrl vs. BCP-ALL ctrl	0.3677	
AML ctrl vs. T-ALL ctrl	0.7502	
T-ALL ctrl vs. BCP-ALL ctrl	0.4849	
BCP-ALL PHA vs. BCP-ALL ctrl	0.1574	
BCP-ALL PMA+I vs. BCP-ALL ctrl	<0.0001	####
BCP-ALL LPS vs. BCP-ALL ctrl	0.48	
T-ALL PHA vs. T-ALL ctrl	0.2313	
T-ALL PMA+I vs. T-ALL ctrl	0.0642	
T-ALL LPS vs. T-ALL ctrl	0.1574	
AML PHA vs. AML ctrl	0.48	
AML PMA+I vs. AML ctrl	0.2313	
AML LPS vs. AML ctrl	0.595	
BCP-ALL PHA vs. T-ALL PHA	0.1574	
BCP-ALL PMA+I vs. T-ALL PMA+I	0.636	
BCP-ALL LPS vs. T-ALL LPS	0.0397	*
BCP-ALL PHA vs. AML PHA	0.3487	
BCP-ALL PMA+I vs. AML PMA+I	0.7502	
BCP-ALL LPS vs. AML LPS	0.2688	
T-ALL PHA vs. AML PHA	0.6897	
T-ALL PMA+I vs. AML PMA+I	0.6205	
T-ALL LPS vs. AML LPS	0.48	

VEGF

All cases

Compared groups	Adjusted p value	Significance level
AML ctrl vs. BCP-ALL ctrl	0.0971	
AML ctrl vs. T-ALL ctrl	0.4071	
T-ALL ctrl vs. BCP-ALL ctrl	0.4408	
BCP-ALL PHA vs. BCP-ALL ctrl	0.2239	
BCP-ALL PMA+I vs. BCP-ALL ctrl	<0.0001	####
BCP-ALL LPS vs. BCP-ALL ctrl	0.2594	
T-ALL PHA vs. T-ALL ctrl	0.1247	

T-ALL PMA+I vs. T-ALL ctrl	0.0563	
T-ALL LPS vs. T-ALL ctrl	0.1666	
AML PHA vs. AML ctrl	0.2272	
AML PMA+I vs. AML ctrl	0.0563	
AML LPS vs. AML ctrl	0.3128	
BCP-ALL PHA vs. T-ALL PHA	0.0563	
BCP-ALL PMA+I vs. T-ALL PMA+I	0.7103	
BCP-ALL LPS vs. T-ALL LPS	0.0727	
BCP-ALL PHA vs. AML PHA	0.0135	*
BCP-ALL PMA+I vs. AML PMA+I	0.374	
BCP-ALL LPS vs. AML LPS	0.023	*
T-ALL PHA vs. AML PHA	0.5613	
T-ALL PMA+I vs. AML PMA+I	0.4071	
T-ALL LPS vs. AML LPS	0.5992	

PDGF

All cases

Compared groups	Adjusted p value	Significance level
AML ctrl vs. BCP-ALL ctrl	0.5542	
AML ctrl vs. T-ALL ctrl	0.6294	
T-ALL ctrl vs. BCP-ALL ctrl	0.3198	
BCP-ALL PHA vs. BCP-ALL ctrl	0.5465	
BCP-ALL PMA+I vs. BCP-ALL ctrl	<0.0001	####
BCP-ALL LPS vs. BCP-ALL ctrl	0.8268	
T-ALL PHA vs. T-ALL ctrl	0.3374	
T-ALL PMA+I vs. T-ALL ctrl	0.103	
T-ALL LPS vs. T-ALL ctrl	0.2719	
AML PHA vs. AML ctrl	0.3198	
AML PMA+I vs. AML ctrl	0.0896	
AML LPS vs. AML ctrl	0.4331	
BCP-ALL PHA vs. T-ALL PHA	0.0535	
BCP-ALL PMA+I vs. T-ALL PMA+I	0.4658	
BCP-ALL LPS vs. T-ALL LPS	0.0104	*
BCP-ALL PHA vs. AML PHA	0.1234	
BCP-ALL PMA+I vs. AML PMA+I	0.6294	
BCP-ALL LPS vs. AML LPS	0.1079	
T-ALL PHA vs. AML PHA	0.6489	
T-ALL PMA+I vs. AML PMA+I	0.6489	
T-ALL LPS vs. AML LPS	0.497	
