# Supplementary material for: Profiles of Growth Factors Secreted by In Vitro-Stimulated Paediatric Acute Leukaemia Blasts of Myeloid and Lymphoid Origin
Source: Int J Mol Sci. 2026 Jan 17;27(2):933. doi: 10.3390/ijms27020933 (PMC12841711; doi:10.3390/ijms27020933)
Supplement: Supplementary file 1 [file ijms-27-00933-s001.zip › Supplementary Table S2.pdf]

Supplementary Table S2. Adjusted p' values after exclusion of cases with 2 replicates, after application of the Benjamini, Krieger and Yekutieli procedure to control the false discovery rate associated with multiple comparisons of concentrations of the examined growth factors in cultures of leukaemic blasts of BCP-ALL, T-ALL and AML patients. Significant differences between stimulated vs. control conditions were marked with hash symbols (# p<0.05, ### p<0.001, #### p<0.0001). Significant differences between different leukaemia types were marked with asterisks (* p<0.05, ** p<0.01). Parts of the table containing the data for specific growth factor were truncated to only show 21 essential comparisons used for drawing conclusions of the performed study.

GM-CSF

Excluded cases with 2 replicates

Compared groups	Adjusted p' value	Significance level
AML ctrl vs. BCP-ALL ctrl	0.0192	*
AML ctrl vs. T-ALL ctrl	0.0861	
T-ALL ctrl vs. BCP-ALL ctrl	0.5073	
BCP-ALL PHA vs. BCP-ALL ctrl	0.0298	#
BCP-ALL PMA+I vs. BCP-ALL ctrl	<0.0001	####
BCP-ALL LPS vs. BCP-ALL ctrl	0.1757	
T-ALL PHA vs. T-ALL ctrl	0.3158	
T-ALL PMA+I vs. T-ALL ctrl	0.0005	###
T-ALL LPS vs. T-ALL ctrl	0.1025	
AML PHA vs. AML ctrl	0.3246	
AML PMA+I vs. AML ctrl	0.0207	#
AML LPS vs. AML ctrl	0.3909	
BCP-ALL PHA vs. T-ALL PHA	0.6267	
BCP-ALL PMA+I vs. T-ALL PMA+I	0.192	
BCP-ALL LPS vs. T-ALL LPS	0.1227	
BCP-ALL PHA vs. AML PHA	0.0467	*
BCP-ALL PMA+I vs. AML PMA+I	0.0539	
BCP-ALL LPS vs. AML LPS	0.0213	*
T-ALL PHA vs. AML PHA	0.1037	
T-ALL PMA+I vs. AML PMA+I	0.3698	
T-ALL LPS vs. AML LPS	0.3246	

G-CSF

Excluded cases with 2 replicates

Compared groups	Adjusted p value	Significance level
AML ctrl vs. BCP-ALL ctrl	0.0028	**
AML ctrl vs. T-ALL ctrl	0.4264	
T-ALL ctrl vs. BCP-ALL ctrl	0.0788	
BCP-ALL PHA vs. BCP-ALL ctrl	0.04	#
BCP-ALL PMA+I vs. BCP-ALL ctrl	<0.0001	####
BCP-ALL LPS vs. BCP-ALL ctrl	0.0655	
T-ALL PHA vs. T-ALL ctrl	0.2669	
T-ALL PMA+I vs. T-ALL ctrl	0.1868	
T-ALL LPS vs. T-ALL ctrl	0.2102	
AML PHA vs. AML ctrl	0.6606	
AML PMA+I vs. AML ctrl	0.4224	

AML LPS vs. AML ctrl	0.6606	
BCP-ALL PHA vs. T-ALL PHA	0.0538	
BCP-ALL PMA+I vs. T-ALL PMA+I	0.731	
BCP-ALL LPS vs. T-ALL LPS	0.0286	*
BCP-ALL PHA vs. AML PHA	0.0319	*
BCP-ALL PMA+I vs. AML PMA+I	0.5339	
BCP-ALL LPS vs. AML LPS	0.0237	*
T-ALL PHA vs. AML PHA	0.7381	
T-ALL PMA+I vs. AML PMA+I	0.6946	
T-ALL LPS vs. AML LPS	0.7407	

b-FGF

Excluded cases with 2 replicates

Compared groups	Adjusted p value	Significance level
AML ctrl vs. BCP-ALL ctrl	0.4744	
AML ctrl vs. T-ALL ctrl	0.8024	
T-ALL ctrl vs. BCP-ALL ctrl	0.5601	
BCP-ALL PHA vs. BCP-ALL ctrl	0.2388	
BCP-ALL PMA+I vs. BCP-ALL ctrl	<0.0001	####
BCP-ALL LPS vs. BCP-ALL ctrl	0.6125	
T-ALL PHA vs. T-ALL ctrl	0.2388	
T-ALL PMA+I vs. T-ALL ctrl	0.0718	
T-ALL LPS vs. T-ALL ctrl	0.207	
AML PHA vs. AML ctrl	0.5275	
AML PMA+I vs. AML ctrl	0.2388	
AML LPS vs. AML ctrl	0.6125	
BCP-ALL PHA vs. T-ALL PHA	0.207	
BCP-ALL PMA+I vs. T-ALL PMA+I	0.739	
BCP-ALL LPS vs. T-ALL LPS	0.0528	
BCP-ALL PHA vs. AML PHA	0.3968	
BCP-ALL PMA+I vs. AML PMA+I	0.7335	
BCP-ALL LPS vs. AML LPS	0.3099	
T-ALL PHA vs. AML PHA	0.7128	
T-ALL PMA+I vs. AML PMA+I	0.6287	
T-ALL LPS vs. AML LPS	0.5311	

VEGF

Excluded cases with 2 replicates

Compared groups	Adjusted p value	Significance level
AML ctrl vs. BCP-ALL ctrl	0.112	
AML ctrl vs. T-ALL ctrl	0.4182	
T-ALL ctrl vs. BCP-ALL ctrl	0.4809	
BCP-ALL PHA vs. BCP-ALL ctrl	0.224	
BCP-ALL PMA+I vs. BCP-ALL ctrl	<0.0001	####
BCP-ALL LPS vs. BCP-ALL ctrl	0.3732	
T-ALL PHA vs. T-ALL ctrl	0.1123	

T-ALL PMA+I vs. T-ALL ctrl	0.0525	
T-ALL LPS vs. T-ALL ctrl	0.1541	
AML PHA vs. AML ctrl	0.2259	
AML PMA+I vs. AML ctrl	0.0588	
AML LPS vs. AML ctrl	0.3294	
BCP-ALL PHA vs. T-ALL PHA	0.0779	
BCP-ALL PMA+I vs. T-ALL PMA+I	0.697	
BCP-ALL LPS vs. T-ALL LPS	0.0714	
BCP-ALL PHA vs. AML PHA	0.0228	*
BCP-ALL PMA+I vs. AML PMA+I	0.4182	
BCP-ALL LPS vs. AML LPS	0.0257	*
T-ALL PHA vs. AML PHA	0.5826	
T-ALL PMA+I vs. AML PMA+I	0.4349	
T-ALL LPS vs. AML LPS	0.6149	

PDGF

Excluded cases with 2 replicates

Compared groups	Adjusted p value	Significance level
AML ctrl vs. BCP-ALL ctrl	0.4504	
AML ctrl vs. T-ALL ctrl	0.6213	
T-ALL ctrl vs. BCP-ALL ctrl	0.236	
BCP-ALL PHA vs. BCP-ALL ctrl	0.412	
BCP-ALL PMA+I vs. BCP-ALL ctrl	<0.0001	####
BCP-ALL LPS vs. BCP-ALL ctrl	0.7585	
T-ALL PHA vs. T-ALL ctrl	0.3581	
T-ALL PMA+I vs. T-ALL ctrl	0.1001	
T-ALL LPS vs. T-ALL ctrl	0.2711	
AML PHA vs. AML ctrl	0.3351	
AML PMA+I vs. AML ctrl	0.0901	
AML LPS vs. AML ctrl	0.4484	
BCP-ALL PHA vs. T-ALL PHA	0.0628	
BCP-ALL PMA+I vs. T-ALL PMA+I	0.4288	
BCP-ALL LPS vs. T-ALL LPS	0.008	**
BCP-ALL PHA vs. AML PHA	0.1498	
BCP-ALL PMA+I vs. AML PMA+I	0.5977	
BCP-ALL LPS vs. AML LPS	0.0901	
T-ALL PHA vs. AML PHA	0.6435	
T-ALL PMA+I vs. AML PMA+I	0.6435	
T-ALL LPS vs. AML LPS	0.4611	
