# Supplementary material for: Profiles of Growth Factors Secreted by In Vitro-Stimulated Paediatric Acute Leukaemia Blasts of Myeloid and Lymphoid Origin
Source: Int J Mol Sci. 2026 Jan 17;27(2):933. doi: 10.3390/ijms27020933 (PMC12841711; doi:10.3390/ijms27020933)
Supplement: Supplementary file 1 [file ijms-27-00933-s001.zip › Supplementary Table S3.pdf]

Supplementary Table S3. Post-hoc power analysis calculated at fixed effect size d=0.50, for the three groups of comparisons performed between groups of different size (n).

Compared groups	Post-hoc power [%]
n=10 vs. n=10	
AML ctrl vs. T-ALL ctrl	28.5
T-ALL PHA vs. T-ALL ctrl	
T-ALL PMA+I vs. T-ALL ctrl	
T-ALL LPS vs. T-ALL ctrl	
AML PHA vs. AML ctrl	
AML PMA+I vs. AML ctrl	
AML LPS vs. AML ctrl	
T-ALL PHA vs. AML PHA	
T-ALL PMA+I vs. AML PMA+I	
T-ALL LPS vs. AML LPS	
n=10 vs. n=46	
AML ctrl vs. BCP-ALL ctrl	40.9
T-ALL ctrl vs. BCP-ALL ctrl	
BCP-ALL PHA vs. T-ALL PHA	
BCP-ALL PMA+I vs. T-ALL PMA+I	
BCP-ALL LPS vs. T-ALL LPS	
BCP-ALL PHA vs. AML PHA	
BCP-ALL PMA+I vs. AML PMA+I	
BCP-ALL LPS vs. AML LPS	
n=46 vs. n=46	
BCP-ALL PHA vs. BCP-ALL ctrl	76.9
BCP-ALL PMA+I vs. BCP-ALL ctrl	
BCP-ALL LPS vs. BCP-ALL ctrl	
